# Automated modeling to high level-of-detail composite object using spatial BIM objects and properties

**DOI:** 10.1038/s41598-025-32433-9

**Published:** 2025-12-26

**Authors:** Jin-Kook Lee, Dahngyu Cho, Youngchae Kim, Yunjeong Mo

**Affiliations:** 1 Dept of Interior Architecture and Built Environment, Yonsei University, 50 Yonsei-ro, Seodaemun-gu, Seoul, Republic of Korea; 2 Dept of Civil, Construction, and Environmental Engineering, Iowa State University, 813 Bissell Road, Ames, IA 50011 USA

**Keywords:** BIM, Composite object, Space object, Parametric modeling, Detailed modeling in automation, Engineering, Mathematics and computing

## Abstract

Building Information Modeling (BIM) procedures for geometric and information modeling are laborious and time-intensive. Traditional methods focus on individual objects, with designers manually modeling each item. This paper presents an approach to high Level of Development (LoD) indoor spatial modeling using space-centered BIM composite objects. By automatically positioning these composite objects within a space, designers can achieve more efficient indoor design modeling early on. The study defines BIM composite objects and demonstrates a visual-language-based parametric modeling process integrated into BIM authoring tools. The process involves inputting data, selecting and placing interior design objects, and deriving the indoor layout. The feasibility of the space-centered design approach is demonstrated in interior architecture, showcasing its potential to reduce inefficiencies and enhance the modeling process, especially in the initial design stage. This approach not only streamlines the early design phase but also reduces inefficiencies across a wide range of applications. The demonstration is implemented and presented in the paper.

## Introduction

### Research motivation

 Indoor space modeling involves creating detailed three-dimensional (3D) models of indoor spaces during the final design stage^1,2^. In this phase, interior design objects—both functional and decorative (e.g., furniture components)—are selected and positioned to enhance visual appeal after partitioning the space with fundamental components such as walls and floors^3^. The selection and arrangement of these elements depend on the specific characteristics of the target space, necessitating the collection of comprehensive information and design requirements to develop an optimal arrangement plan^2,4^.

The traditional “object-oriented” approach requires manually placing each object in the environment^5^. This approach is both labor-intensive and inefficient, as it demands that each object be individually loaded and manually positioned, which may result in ergonomically suboptimal arrangements.

The impact of Artificial Intelligence (AI) and automation on indoor architecture has spurred discussions on redefining the role of interior designers by shifting routine, repetitive tasks to machines, thereby freeing designers to focus on creative aspects^6,7^. In the AI era, machines are predicted to handle repetitive tasks efficiently, while designers concentrate on more innovative work^8–17^.

**Fig. 1:**
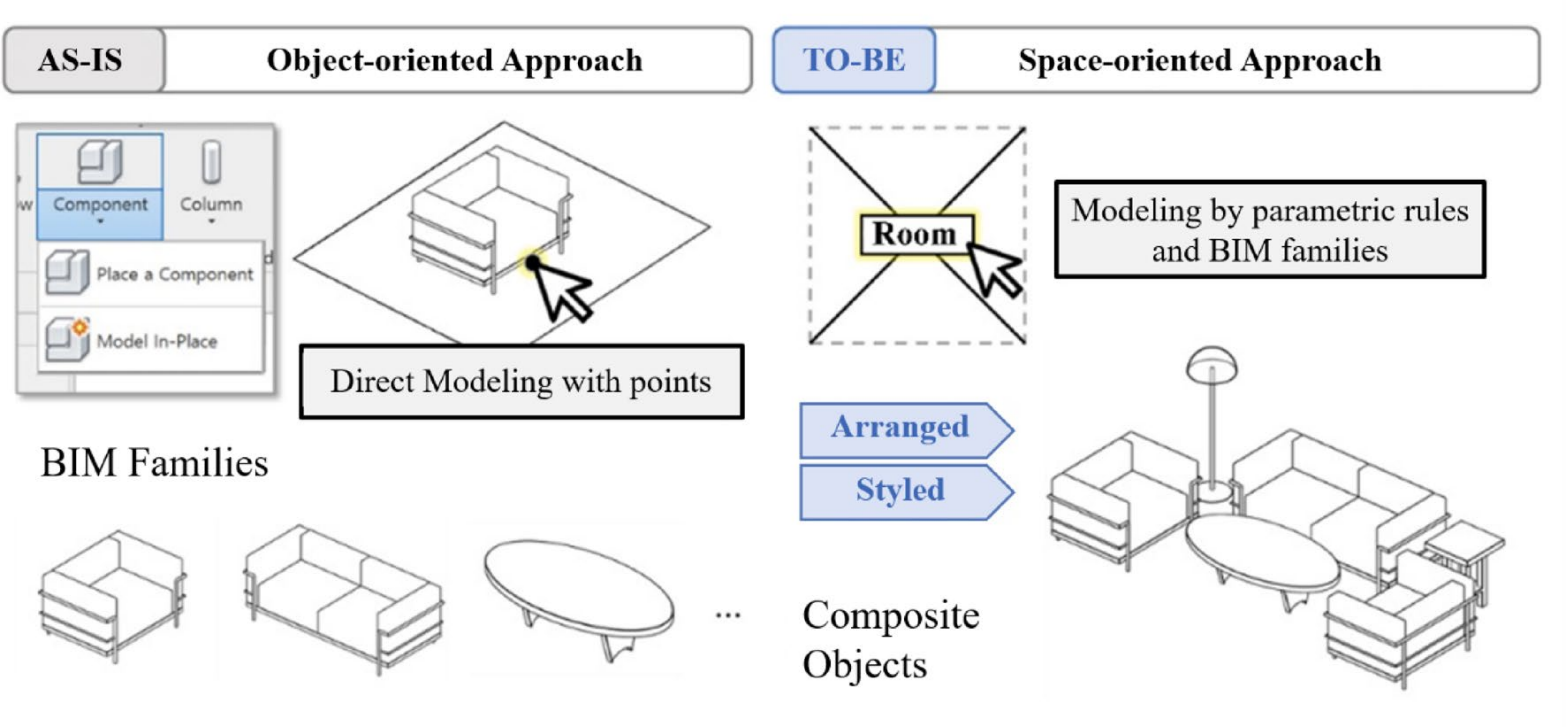
Overview of space-oriented approach. This contrasts the traditional Object-oriented Approach (As-Is) with the proposed method (To-Be), where selecting a ‘Room’ object parametrically generates a ‘Composite Object’ group.

In response to these trends, this study aims to develop a systematic “space-oriented” approach, treating space as the primary entity and focusing on object placement within it^18^. We propose utilizing space object attributes in Building Information Modeling (BIM) as variables to design space and group them into a BIM composite object—a collection of interior design elements arranged within a specific space according to predefined parametric rules (Fig. 1). This strategy streamlines redundant tasks and promotes a more efficient arrangement of interior design elements^19–22^.

In Seoul, the demand for affordable, immediately available housing for socially vulnerable groups is rising. According to the Seoul Housing Portal^23^, urban lifestyle housing is emerging as a viable alternative to meet the growing demand for 1–2 person households and ensure housing stability. Yet, current methods for visualizing design requirements are cumbersome and time-intensive, impeding the swift delivery of housing^24^. The proposed “space-oriented” approach is expected to simplify the design process and accelerate housing supply by automating the placement of interior design elements using BIM technology, potentially addressing housing supply challenges in Seoul and improving housing stability for vulnerable populations.

### Research purpose and overall process

This study aims to simplify indoor space modeling by defining a space-centered BIM composite object that integrates detailed design components such as interior design elements. Additionally, it develops a parametric placement methodology for these composite objects based on design guidelines and spatial parameters. The objectives are: (1) to define interior composite objects, (2) to establish interior design rules, and (3) to implement composite objects within a BIM tool.

A building consists of several spaces, each serving specific functions; similarly, a space-centered composite object refers to a combination of furniture objects that create functional spaces. By applying input variables related to the target space and adhering to a design rule dataset, the space can be parametrically designed in various ways. This study follows the guidelines of the Korea Land and Housing Corporation and creates an algorithmically generated rule dataset that captures general furniture component arrangement patterns for indoor spaces.

**Fig. 2:**
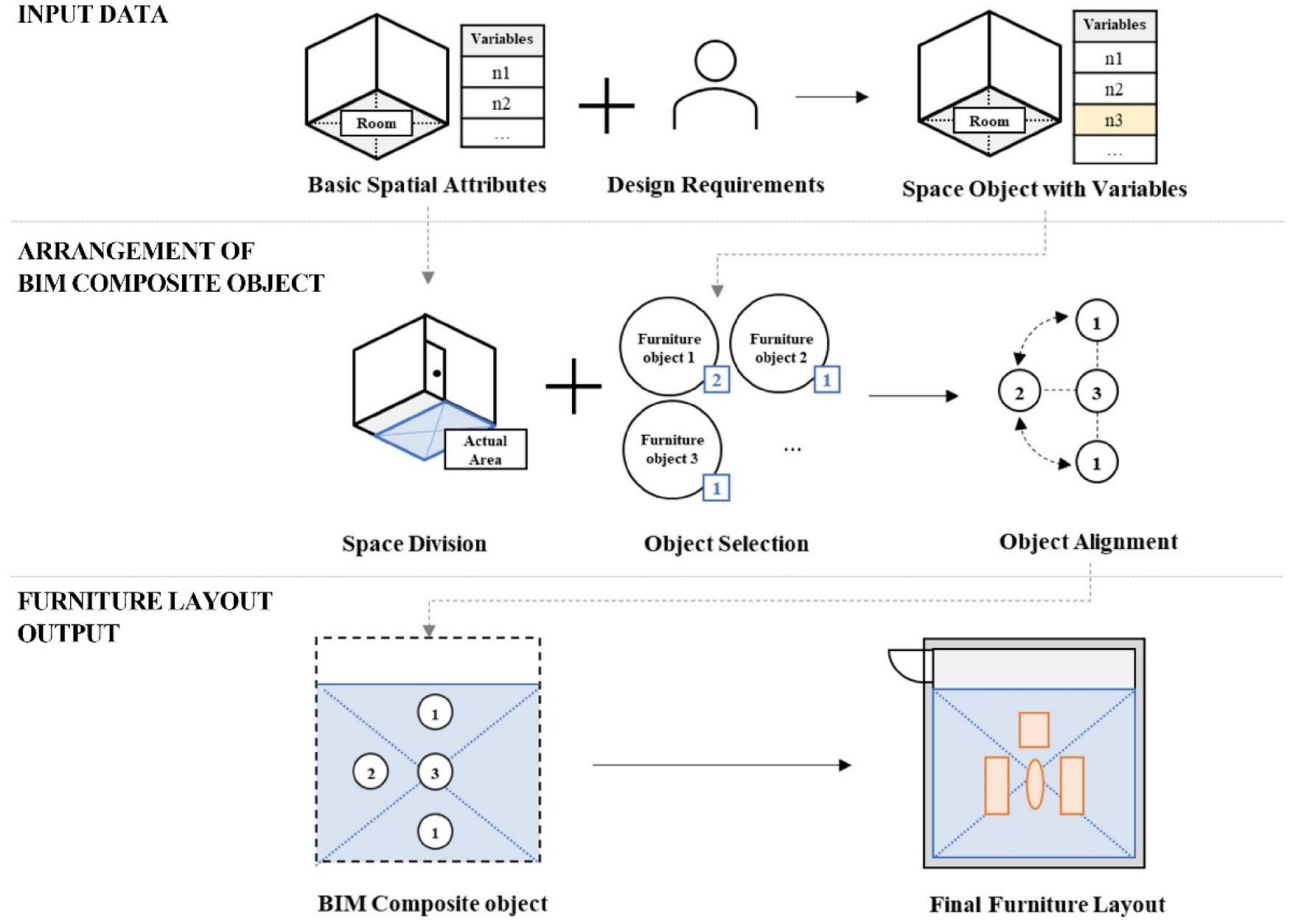
Space modeling process using BIM composite objects.

This research introduces a BIM approach that employs composite object-based modeling with spatial objects and a rule-based parametric placement strategy during the early design stages. Residential spaces were selected due to their distinct functions, with the living room serving as the primary test case in the prototype. Suitable furniture for each space is chosen based on function, and the number of objects is adjusted according to the number of users. For multifunctional spaces, object arrangements vary accordingly. The furniture layouts are derived from existing designs^25,26^. The prototype was developed using Autodesk Revit^27^ and Dynamo^28^, following three stages (Fig. 2): (1) data input from BIM space objects and users, (2) arrangement of BIM composite objects, (3) output of furniture layouts in response to external stimuli and a fixed layout.

The paper is organized as follows: Sect. 2 reviews existing studies on furniture arrangement modeling and design guidelines for residential spaces; Sect. 3 defines BIM composite objects and outlines their parametric variables; Sect. 4 details their application in residential space modeling; Sect. 5 presents a prototype showcasing living room designs with various scenarios; and finally, Sect. 6 discusses the findings and future directions.

The main contributions of this study are as follows. First, we propose the concept of the ‘BIM Composite Object,’ a functional unit of space, to simplify the design process. Second, we present a methodology for converting existing design guidelines into parametric rules, enabling the use of qualitative knowledge as quantitative data. This approach is fundamentally different from previous studies in the computer graphics and automated layout domain, such as those by Merrell et al.^29^ and Wang et al.^18^, which focused on optimizing the relationships between individual objects for visual scene generation. Our method enables modeling in ‘spatial sets’ that are directly linked to BIM data in the early design stages, thereby maximizing efficiency.

Residential space modeling with BIM composite objects leverages accumulated knowledge in interior design along with computational design techniques. The main terminologies used in this research are explained below:

- *BIM Composite Object*: A BIM composite object refers to a collection of pre-defined individual furniture objects selected based on the characteristics of the target space.
- *Parametric Modeling*: Parametric design is a process that allows changes to a model’s properties, such as its shapes and relationships, when various aspects are modified. This approach is often implemented using programming scripts that define the dimensions or attributes of the model.
- *Property*: A property is a set of parameters that define a particular aspect of an object. These parameters may include typical actions, materials used, and contextual characteristics that influence the behavior or appearance of the object.
- *Relation*: The established connections between one object and another.
- *Rule*: Parametric rules build relationships among multiple design elements. The same algorithm should be applied throughout the project to ensure that modifications in specific elements trigger the necessary adjustments across the design.
- *Constraint*: A constraint that design alternatives must satisfy, confining them to specified value ranges. An unconstrained study may yield unrealistic or impractical results.
- *Variable*: A value that can change during the generation of outcomes. In this study, the variables include space area, space type, number of users, and space function.

## Background

### Parametric modeling in design stage

Object-based parametric modeling employs rules and parameters to define objects and their properties, both geometric and non-geometric. These rules and parameters enable objects to automatically update in response to changing conditions or user input. Geometric relationships, such as angles and distances, as well as rules like “attached to” or “parallel to”, can be specified to establish relationships between objects and their surfaces. Within the BIM environment, custom parametric objects are encouraged, allowing users to define new objects either as solid geometric models or as parametric object families. These object families include rules and parameters that facilitate self-updating behaviors.

In the interior architecture design stage, the relational structure is particularly critical in parametric modeling, alongside the growing emphasis on shape parametric modeling. For example, when a door is installed within a wall, a new relationship is formed between the door and the wall. Although the relationships between building objects used during the construction stage have been extensively studied in parametric modeling, the relationships among interior design objects have often been overlooked within the same BIM parametric context^30^. This paper, therefore, focuses on the relationships among interior design objects and their placement, employing pre-defined parametric rules specifically designed for positioning these elements.

### Furniture arrangement

The furniture arrangement system is an actively researched topic in the design field, encompassing indoor scene studies, design tools, and rule-checking^16,29,31^. Most studies in this area primarily focus on the semi-automated or automated arrangement of furniture objects within a designated space. These studies use parametric variables and constraints to guide object placement. For example, Merrell et al.^29^ developed a system that employs interior design principles, including density functions, to create layouts that optimize both practical and visual criteria. Practical criteria evaluate the system’s assistance in human activities by considering factors such as clearance, circulation, pairwise relationships, and conversation, while visual criteria focus on the recognition of visual composition by considering aspects like balance, alignment, and emphasis. Similarly, Yu et al.^32^ developed an automated system that generates furniture arrangements for complex indoor spaces, considering critical ergonomic influences. This system accounts for various interacting factors, including spatial relationships within the room, pairwise relationships between furniture objects, and other human factors, to optimize furniture arrangement. Moreover, they introduced a framework for furniture arrangement.

Kán et al.^33^ proposed a framework for arranging furniture in virtual indoor scenes, optimizing position and orientation based on ergonomic, aesthetic, and functional rules outlined in interior design guidelines. The arrangement process considered various properties of furniture objects, such as clearance constraints, the probability of being placed against a wall, possible parent objects, the likelihood of having a parent object, room importance, and desired count. Wang et al.^18^ introduced Plan IT, a system that generates furniture layouts by constructing a relationship graph representing feasible relationships among objects. Their analysis includes relationships such as functional symmetries, support edges, spatial edges, and superstructures, and the system then creates a 3D scene adhering to the plan. Sydora & Stroulia^31^ demonstrated an automated approach for generating alternative interior designs that conform to a set of design rules for rule-checking purposes. The design guidelines incorporate various factors, including clearance, circulation, pairwise relationships, conversation, balance, alignment, and emphasis^34–36^.

**Table 1:** Variable classification for furniture arrangement in related studies (‘-’ denotes not provided).

Information	Merrel et al. (2011)^29^	Yu et al. (2011)^32^	Kan et al. (2017)^33^	Wang et al. (2019)^18^	Sydora et al. (2020)^31^
Arrangement Method	Conversation, balance, alignment, emphasis	Center and orientation, viewing frustum, spatial relationships	Probability of standing against a wall, room importance	Functional symmetries	Conversation, balance, alignment, emphasis
Area	-	Bounding surface	Desired count	-	-
Circulation	Circulation	-	-	-	Circulation
Clearance	Clearance	Accessible space	Clearance constraints	Spatial edges	Clearance
Correlation (weak bond)	Pairwise relationship	Pairwise relationships	-	Superstructures	Pairwise
Hierarchy (strong bond)	-	Hierarchical relationships	Possible parents, probability of having a parent	Support edges	-

The main variables identified in the aforementioned studies are classified into six categories: arrangement method, area, circulation, clearance, correlation, and hierarchy (Table 1). Arrangement method focuses on orientation, functional symmetries, and common placement of furniture objects. Area measures the space required for each object. Circulation considers the movement and flow of people within the space. Clearance refers to the minimum space needed around an object to accommodate human behavior. Correlation, also described as a weak bond, represents a non-obligatory but influential relationship between furniture objects (e.g., a sofa and its side table). Hierarchy denotes a strong, often obligatory, parent-child relationship between furniture objects.

While numerous related studies exist, this research distinguishes itself by grouping diverse interior design objects by specific spaces rather than solely arranging furniture for visualization. To this end, the study constructs composite objects based on predetermined criteria that designers can consistently apply during the design phase.

In summary, while previous studies have primarily focused on the visual and functional optimization of generated layouts, this research is distinguished by its ‘space-centered’ approach. Within the BIM environment, we treat the ‘space’ itself as an object with properties. The entire furniture group (Composite Object) is then parametrically arranged according to this space object. This aims for a more integrated workflow where design changes do not require repositioning individual furniture pieces; instead, the entire layout is automatically updated simply by changing the space’s property values (e.g., number of users, space function).

### Furniture layout guidelines in residential space

**Fig. 3:**
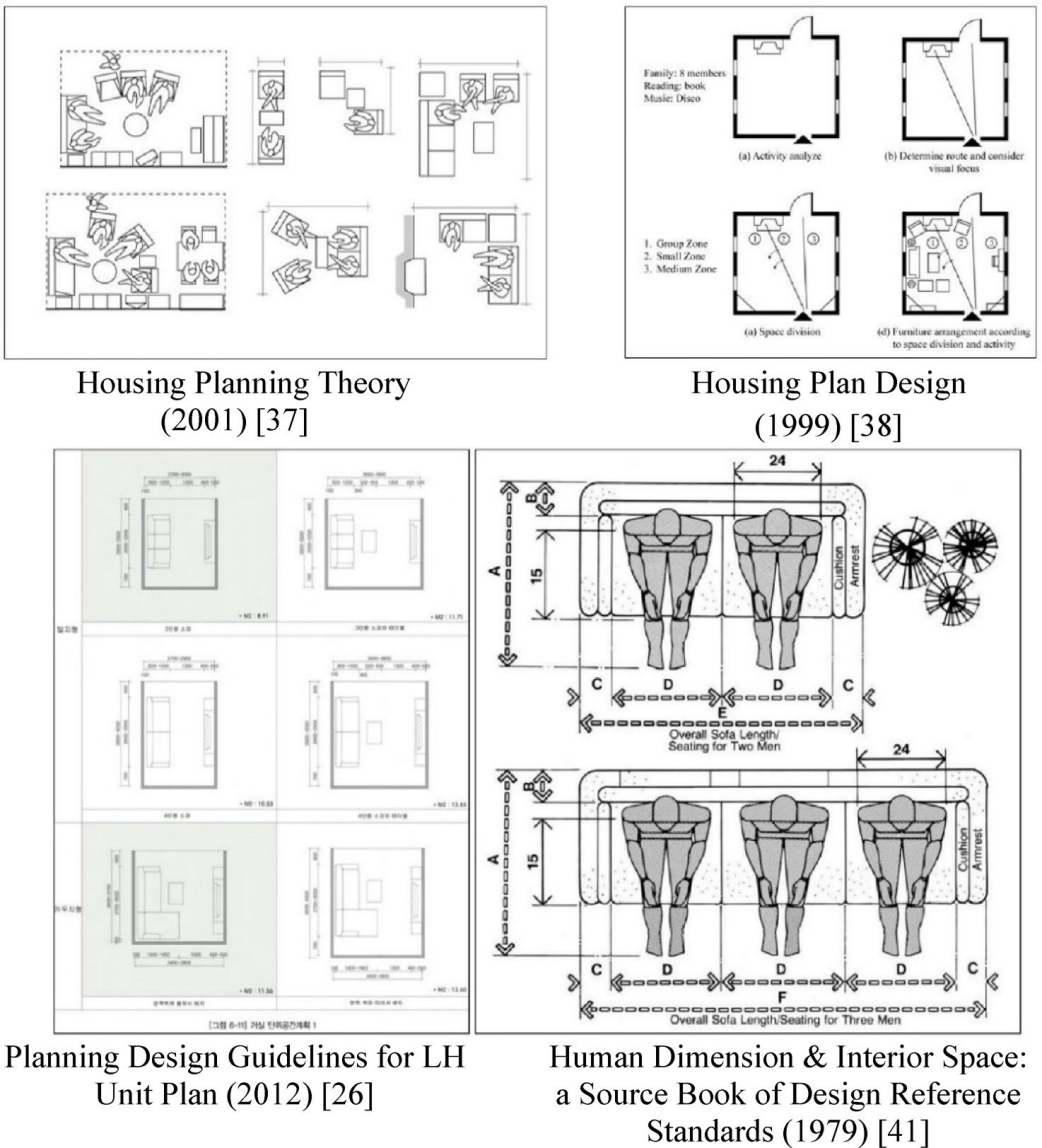
Reference images of furniture design guidelines.

This study consulted several furniture layout manuals to establish guidelines centered on residential spaces. Table 2 provides a comprehensive summary of the definitions, functions, and key design considerations for living rooms from various established guidelines. These guidelines commonly emphasize creating flexible connections, ensuring clear pathways, and arranging furniture to support both primary (e.g., conversation) and secondary (e.g., remote work) functions. Furthermore, ergonomic principles are incorporated to enhance comfort and functionality, as exemplified in Fig. 3.

**Table 2:** A comprehensive summary of the definition, function, and design considerations of the living room in design guidelines.

Design Guidelines	Definition	Function	Design Considerations
Housing Planning Theory (2001)^37^	Shared space for families, public space within the residence, and at the same time, a space that is the center of family life	Facilitates conversation, relaxation, entertainment, personal activities, children’s play, and guest meetings	The living room should accommodate the entire family, considering that each family member has unique needs based on their living conditions.
Housing Plan Design (1999)^38^	The center of family life occupies the largest proportion and area in each room.	Supports TV viewing, facilitates conversation, and provides a clear pathway.	Primarily emphasizes TV viewing and social gatherings while highlighting connectivity between the living room and other rooms.
History of Korean Apartment Housing Planning (1999)^39^	A communal space for family and guests, requiring furnishings such as a sofa, chairs, tables, a TV, and decorative wall elements.	Supports conversation, TV viewing, music listening, indoor games, and provides a clear pathway.	Ensures that furniture placement does not obstruct pathways to other rooms.
Interior Design (1988)^40^	Living rooms are used for a variety of purposes, from formal events to multipurpose spaces such as studios.	Basic functions include conversation and entertainment; expansion functions may include study, office, or music listening areas.	Furniture should be arranged to reflect both primary and secondary functions, with seating configurations adapted for TV viewing or window placement.
Human Dimension & Interior Space: a Source Book of Design Reference Standards (1979)^41^	-	Includes designated rest areas (e.g., sofa, lounge, corner seating), storage solutions (e.g., bar units), and pathways.	Emphasizes ergonomic considerations (e.g., maximum body depth, eye height) and specifies area requirements for each furniture piece.
Planning Design Guidelines for LH Unit Plan (2012)^26^	A space that is both a resting place for families and a space where family conversations take place	Basic functions: family conversation, guest meetings, hobbies, and daily activities; expanded functions: housework, storage, family events, and exercise.	Considers the operational dimensions of the living room and the sizes of furnishings in accordance with Korean housing standards.
The Measure of Man and Woman: Human Factors in design (2001)^43^	A central area for relaxation, socializing, and entertainment, serving as a communal space for family members and guests.	Facilitates social gatherings, entertainment, relaxation, formal receptions, and versatile uses.	Ensures a furniture layout that supports social interaction, allows for easy movement, incorporates well-designed lighting and storage for an organized, clutter-free environment, and maintains nobstructed pathways.
Architects’ Data (2012)^44^	A conventional living room is a shared space that represents the prestigious front of the house for visitors.	Supports multifunctional activities, including communication, play, work, and connections, as well as providing indirect access.	Focuses on the dimensions and arrangement of furniture to ensure universal circulation and accessibility.
Home Office Solutions: How to Set Up an Efficient Workspace Anywhere in Your House (2020)^45^	A living room is a flexible area that can be transformed into a productive home office space, in addition to its traditional roles of relaxation and social gatherings.	Serves as a home office, remote workspace, relaxation hub, and family activity center.	Emphasizes furniture arrangements that delineate work and living zones, incorporates proper lighting for task visibility and concentration, and includes measures for noise control between work and living areas.

### Related applications

**Fig. 4:**
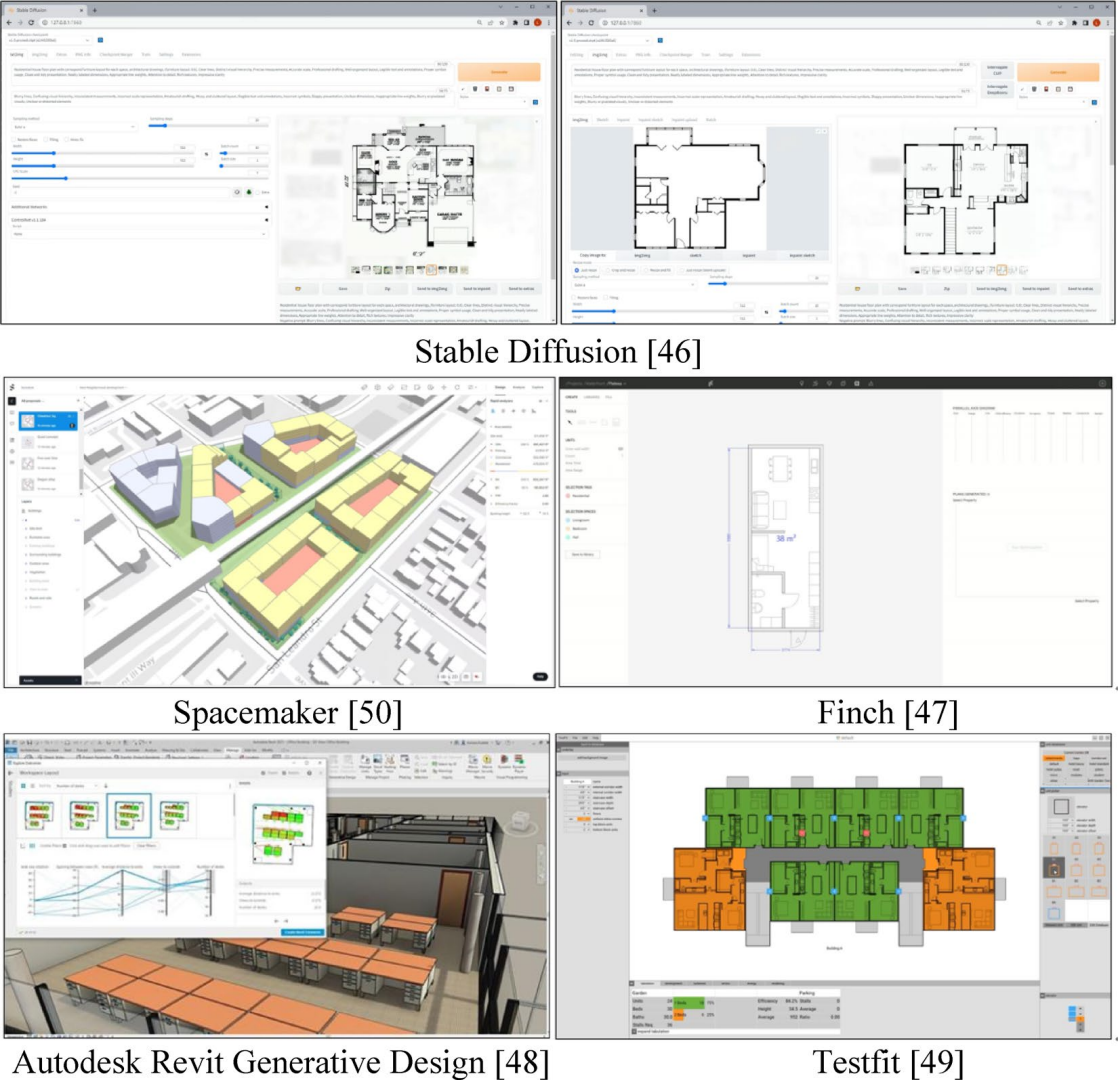
Examples of related applications.

Automating or semi-automating furniture arrangement generation can significantly expedite the design process. Several existing tools such as Stable Diffusion^45^, Finch^46^, Autodesk Revit Generative Design^47^, TestFit^48^, and Spacemaker^49^ provide functionalities in this regard (Fig. 4). Stable Diffusion^45^, an image-generation AI, offers a powerful solution for accelerating architectural design. By leveraging this technology, architects can enhance automation and decision-making capabilities. The AI, driven by customizable inputs, enables the consideration of various factors when generating furniture arrangements. Through iterative exploration, architects can streamline design proposals while maintaining high customization and making data-driven decisions during the early design phase^50^. However, despite its capability to understand relationships among furniture arrangements, spatial functionality, aesthetics, and other factors, there remain technical limitations in directly integrating generated images with actual BIM data. While this technology generates furniture layouts that harmonize with the overall design and optimize functional and visual aspects, it is important to recognize that these are merely visual representations and do not directly integrate with actual design data.

Finch^46^ automates redundant tasks in architecture, guides architects, and facilitates informed decision-making through simulations. Primarily used in early design phases to quickly visualize program distribution and layout, Finch employs a rule-based algorithm with customizable inputs and operates within platforms such as Rhinoceros^51^, Grasshopper^52^, and Autodesk Revit^27^. However, Finch differs from the present study as it focuses mainly on building objects, with interior objects modeled only at an introductory level.

Autodesk Revit Generative Design^47^ generates design alternatives based on user-defined goals, constraints, and inputs, thereby enabling data-driven decision-making. It optimizes these goals by running multiple design generations and refining the outcomes. For instance, in a restaurant layout case study, factors such as table spacing, proximity to exits, outside views, and the number of desks were considered. This tool can focus on individual objects while also performing multi-objective optimization by considering the relationships among multiple objects. While Revit Generative Design is a multi-objective optimization tool requiring users to set goals and constraints, our approach differs by using ‘Composite Objects’ with embedded design knowledge to minimize user input and quickly produce specialized results for specific spatial designs.

TestFit^48^ offers rapid solutions for site plans related to multifamily, hotel, parking, or garden apartments. Utilizing a data-driven approach to smart urban planning, TestFit incorporates user input through co-creation and proprietary algorithms, making it particularly beneficial for cost management in feasibility studies for developers, brokers, and architects.

Similarly, Spacemaker^49^ expedites urban development planning and enhances outcomes by incorporating factors such as user quality of life and sustainability. It allows for rapid creation, optimization, and iteration of design alternatives while considering design standards and diverse datasets including maps, lighting, wind, terrain, traffic, and zoning. The main difference between TestFit, Spacemaker, and this study is their primary focus on urban aspects and space data utilization.

This study, in contrast, proposes generating furniture arrangements that incorporate detailed and qualitative interior design data. While previous research has predominantly addressed architectural design challenges and large-scale cost estimations, the present study aims to bridge the gap by integrating specific interior design knowledge into the process of furniture arrangement generation. By incorporating this specialized knowledge, the study seeks to elevate the quality and functionality of the generated layouts. It considers factors such as spatial organization, user experience, aesthetic preferences, and ergonomic considerations, all of which contribute significantly to creating well-designed and harmonious interior spaces. The objective is to transcend a purely functional and cost-based approach by integrating the expertise and insights of interior designers. This integration strives to produce layouts that not only meet functional requirements but also enhance the overall user experience and aesthetic appeal of the space. By bridging the gap between architecture and interior design, this study aims to equip architects with a comprehensive tool that encompasses both structural aspects and interior design details. The ultimate goal is to empower architects to develop more thoughtful, visually pleasing, and user-centric interior spaces through automated or semi-automated furniture arrangement generation.

## BIM composite object

### Overview

In BIM, building components are represented as objects according to the Industry Foundation Classes (IFC)^53^, an open international standard for BIM data. The IFC provides a data schema that defines objects, including composite objects composed of sub-objects like walls, columns, and doors^54^. This approach offers greater flexibility in modeling complex structures. This study focuses on defining composite objects for interior design, particularly furniture objects and their relationship to the space object. These composite objects are combinations of pre-defined objects that can respond parametrically to external stimuli. They exist within the context of the space object and have constraints influencing their design and placement.

**Fig. 5:**
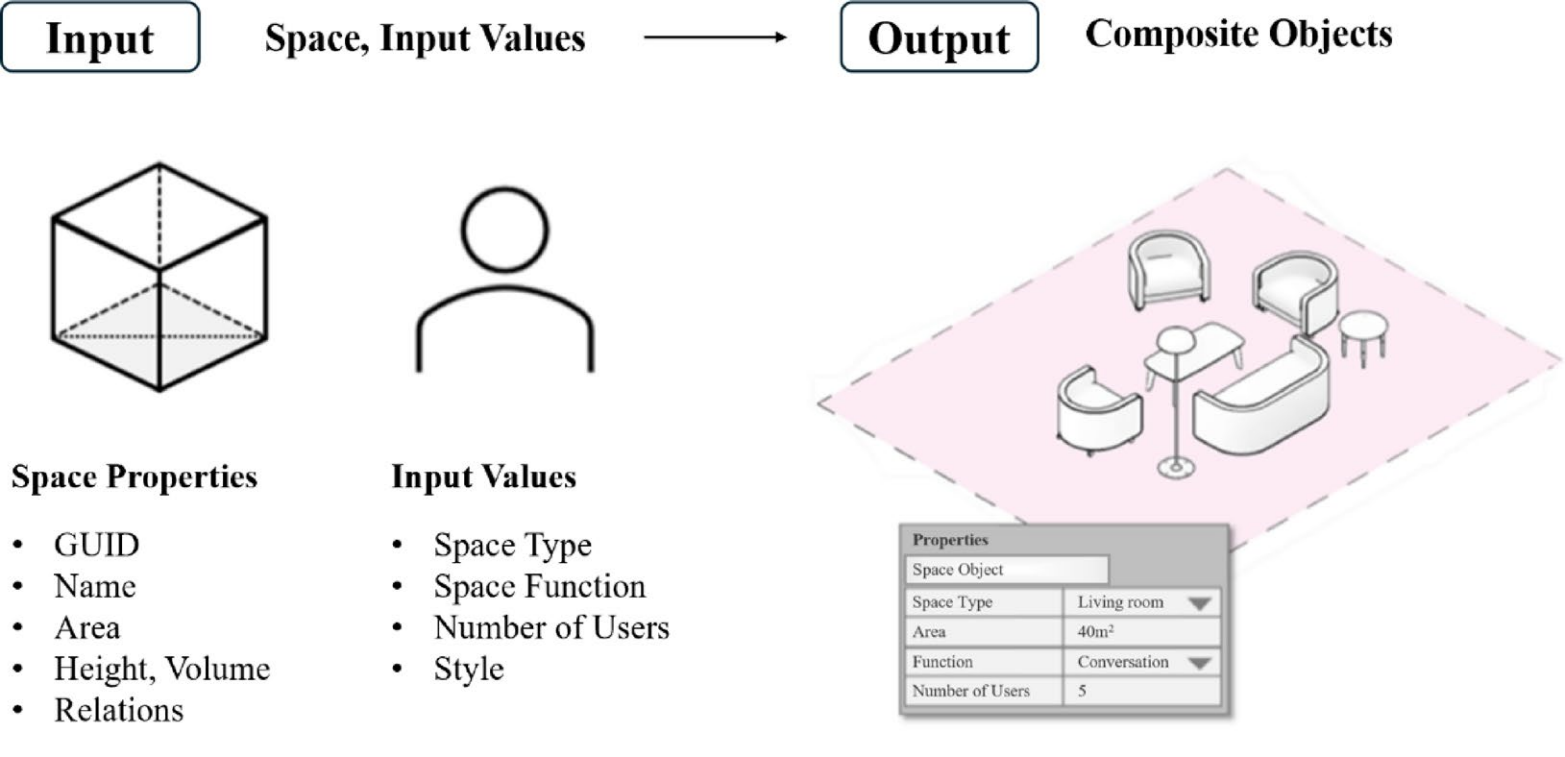
Input and output of the BIM composite object mechanism.

The BIM authoring tool generates space objects using information from surrounding objects. These space objects consist of basic attributes (e.g., Globally Unique Identifier (GUID), Name), calculated attributes (e.g., Net area, volume), and user-input attribute values. Unlike previous studies, the BIM composite object in this research provides a more comprehensive representation by adding interior design objects, transforming it from a theoretical concept into a tangible scene (Fig. 5). This higher level of detail augments the Level of Development (LOD) for the BIM model. By analyzing data and user inputs related to the target space, the necessary interior design objects are identified and integrated, resulting in a filled space recognized as a BIM composite object^55^.

The relationship between the space objects and interior design objects results in variations in the type and arrangement of objects based on changes in spatial attributes. These changes may stem from modifications in basic spatial attributes or user input, such as adjustments to the spatial area or the number of users. Such modifications are implemented in a parametric manner, following pre-defined rules that incorporate existing design guidelines. This approach facilitates the continuous evolution of new designs while maintaining adherence to pre-established design rules.

### Classification of attribute information for composite objects

The IFC standard is crucial to this process as it provides the foundational data structure. Specifically, the IfcSpace entity within the IFC schema offers the basic spatial attributes (e.g., GUID, Name, Net Area) that serve as the initial input for generating our BIM composite objects. This ensures that our methodology can be integrated into a standardized, interoperable BIM workflow.

During the design of indoor spaces, designers consider factors like the size and shape of the space, user requirements, and aesthetic preferences to determine object placement. A similar approach is followed when generating a BIM composite object. The information necessary to interpret the target space and create the composite object includes basic attributes of the space object, user input information, and design rule information derived from design guidelines. By analyzing this information, necessary interior design objects can be identified and parametrically integrated into the space based on predefined rules^56,57^.

**Fig. 6:**
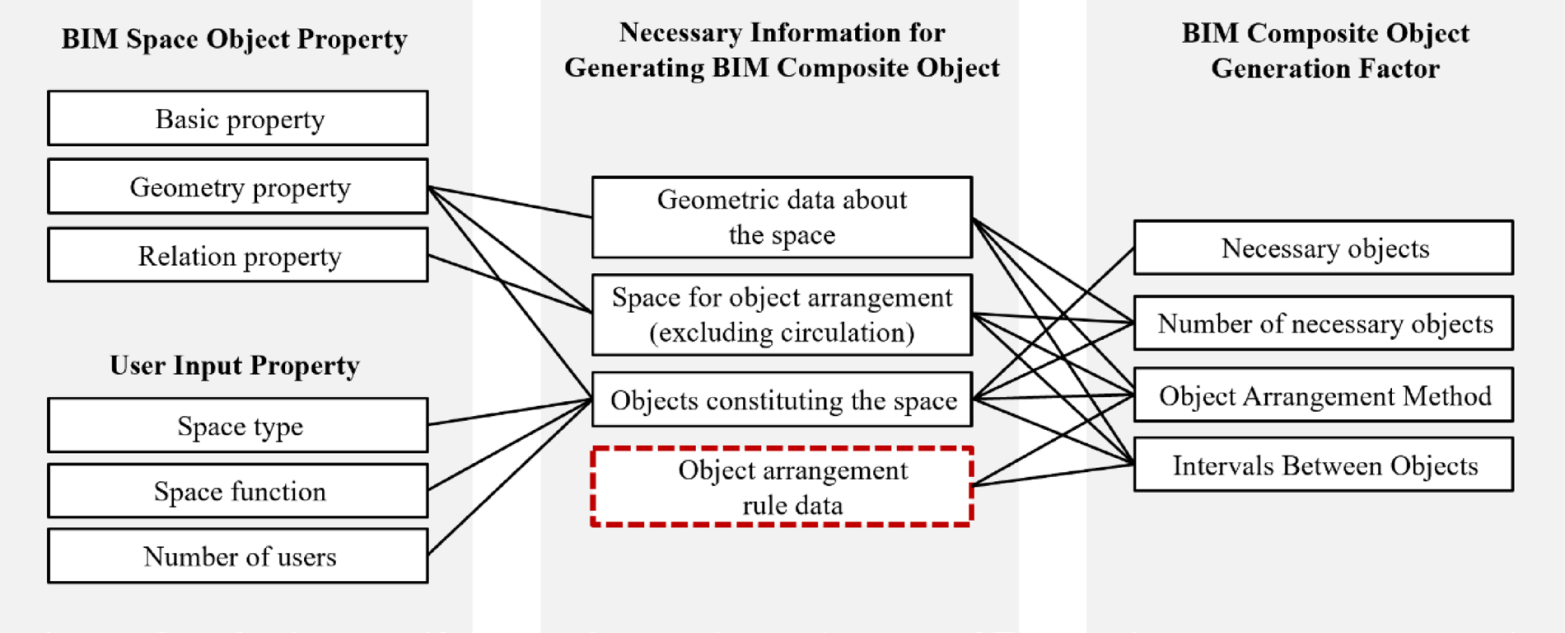
Classification of attributes for generating BIM composite objects. This map shows how properties from BIM Space Object and User Input are translated into Generation Factors. The red-outlined box highlights the core process of translating qualitative guidelines into computable rule data.

Figure 6 illustrates the classification and mapping relationship of this information. In the figure, the red-outlined box is used to emphasize that the process of converting qualitative guidelines into computable rule data is a critical knowledge formalization step in this research. The properties of the BIM Space object and user input serve as sources to generate BIM composite objects. This mapping process revealed the need for additional user input beyond BIM data. Moreover, placing composite objects requires design rule information specific to the space, demanding individual interpretation.

### BIM composite object parametric attributes

The attributes detailed in this section directly correspond to the ‘BIM Composite Object Generation Factors’ identified in Fig. 6. The attributes of BIM composite objects are categorized into two groups: space variables and object properties. Derived from design guidelines, these attributes correspond to relationships and measurements discussed earlier.

#### Translating design guidelines into parametric rules

A key step in our methodology is the formalization of qualitative design knowledge into computable rules. By analyzing established interior design guidelines summarized in Table 2, we derived quantitative parameters that can be implemented in a parametric model. For instance, qualitative requirements such as “comfortable viewing distance” or “unobstructed pathways” were converted into specific metric constraints (e.g., ≥ 2440 mm, ≥ 300 mm) based on standard anthropometric data^41^. These quantitative rules serve as default generative constraints within the algorithm to ensure functional compliance, while still allowing for user refinement in the final arrangement. Table 3 provides the comprehensive set of rules employed in our prototype scenarios.

**Table 3:** Design guideline (qualitative) and its parametric rule (quantitative).

Design Guideline (Qualitative)	Parametric Rule (Quantitative)
Furniture should not obstruct pathways.	The placement of objects is restricted to the ‘Actual Area’ excluding the ‘Circulation Area’.
Arrange seating to facilitate conversation.	Default Angle (Sofa, Chair) = 180° (face-to-face) or 90° (L-shape). (User can refine rotation)
Ensure a comfortable viewing distance for the TV.	Distance (TV, Sofa) ≥ 2440 mm
Ensure minimum circulation space between seating and tables.	Distance (Sofa/Chair, Coffee Table) ≥ 300 mm
Maintain minimum distance between seating furniture.	Distance (Sofa, Chair) ≥ 100 mm
Ensure comfortable passage in workspace areas.	Distance (Desk, Coffee Table) ≥ 1200 mm
Provide clearance for standalone objects.	Clearance (Floor Stand, Surrounding Objects) ≥ 100 mm

#### Host object selection logic

The host object serves as the anchor point in the arrangement process, making its selection logic critical. This logic directly utilizes several object properties detailed below (e.g., movability, hierarchy). Our study determines the host object through the following sequential process:

1. Prioritize Fixed Objects: If an object with a ‘Fixed’ movability property (e.g., a TV, built-in furniture) exists, it is selected as the host. If multiple ‘Fixed’ objects exist, the logic proceeds to the next criterion (Object Hierarchy) to select the one with the highest number of hierarchical connections.
2. Evaluate Object Hierarchy: If no fixed objects are present, the object that is most likely to be surrounded by others (e.g., a coffee table between sofas) is chosen as the host based on the number of hierarchical connections.
3. Compare Movability: If the number of connections is equal, the object with the lowest movability (semi-fixed > movable) is selected to ensure layout stability.

#### Detailed parametric attributes

Based on the aforementioned process, the following variables and properties are defined.

Space variables used for managing the space are:

- Space Area: A basic geometric property calculated in the BIM tool as Net area.
- Space Type: Specified by the user to indicate the desired room type from a predefined list (e.g., living room, dining room, kitchen, bathroom, bedroom).
- Space Function: A user-input variable specifying intended activities in the space, provided as a predefined list specific to the space (e.g., conversation, watching TV, hobby space for a living room).
- Number of Users: Determines occupancy, impacting the number of required furniture objects.

While space variables may change due to external stimuli, object properties remain fixed:

- Object Area: The minimum required area for each object within a specific room.
- Object Hierarchy: Depicts the relationship between objects in terms of host and surroundings. Each object is assigned a probability of being a host when connected to another. The host object is positioned first, and surrounding objects are placed while adhering to clearance constraints.
- Object Movability: Determined by typical location and customs, categorized as fixed, semi-fixed, movable, or mobile. Objects with lower movability levels are more likely to be the host object.
- Object Clearance Constraints: The minimum space required around furniture for human use, defined for the front, back, left, and right sides. Values are based on previous research^29^.
- Probability of Locating Near the Wall: Indicates the likelihood of an object being positioned close to a wall. If the probability is low, the object is placed near the center of the space.

## BIM composite object-based residential space modeling

### Overview of BIM composite object-based residential space modeling

**Fig. 7:**
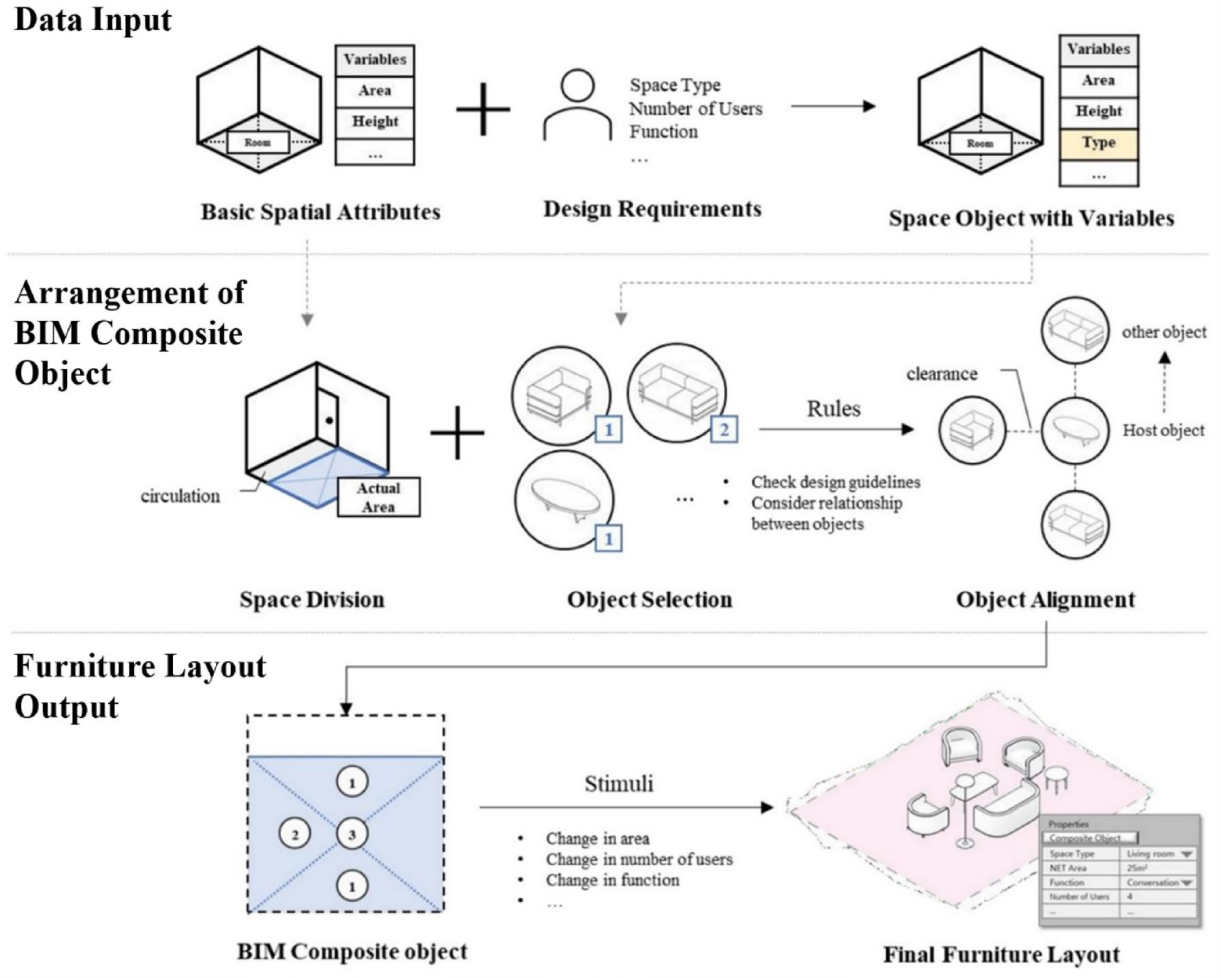
Utilization of BIM composite objects in the residential space modeling process. A diagram of the three-phase process: (1) Data Input, combining ‘Basic Spatial Attributes’ with ‘Design Requirements’; (2) Arrangement of BIM Composite Object, involving ‘Space Division’, ‘Object Selection’, and ‘Object Alignment’; (3) Furniture Layout Output, generating the final layout which can be updated by new stimuli.

This study proposes a process for generating furniture layouts using BIM composite objects, represented as a parametric model with space variables and object properties as major attributes using Autodesk Revit and Dynamo. The process aims to arrange composite objects based on space variables and configure them to respond to external stimuli with three main phases: Data Input, Arrangement of BIM Composite Object, and Furniture Layout Output, as illustrated in Fig. 7. Each phase includes multiple modules that perform specific operations, and the specific steps are as follows.

In ‘Data Input’ phase, the BIM tool’s basic spatial attributes such as GUID, Net Area, and topology data, along with user attributes such as space type, space function, and the number of users, are received. These attributes are used as space variables and can be altered by external stimuli. At the end of this phase, a space object is created, such as a 25m^2^ living room for 4 people with the main function of conversation, which has the defined variables.

**Fig. 8:**
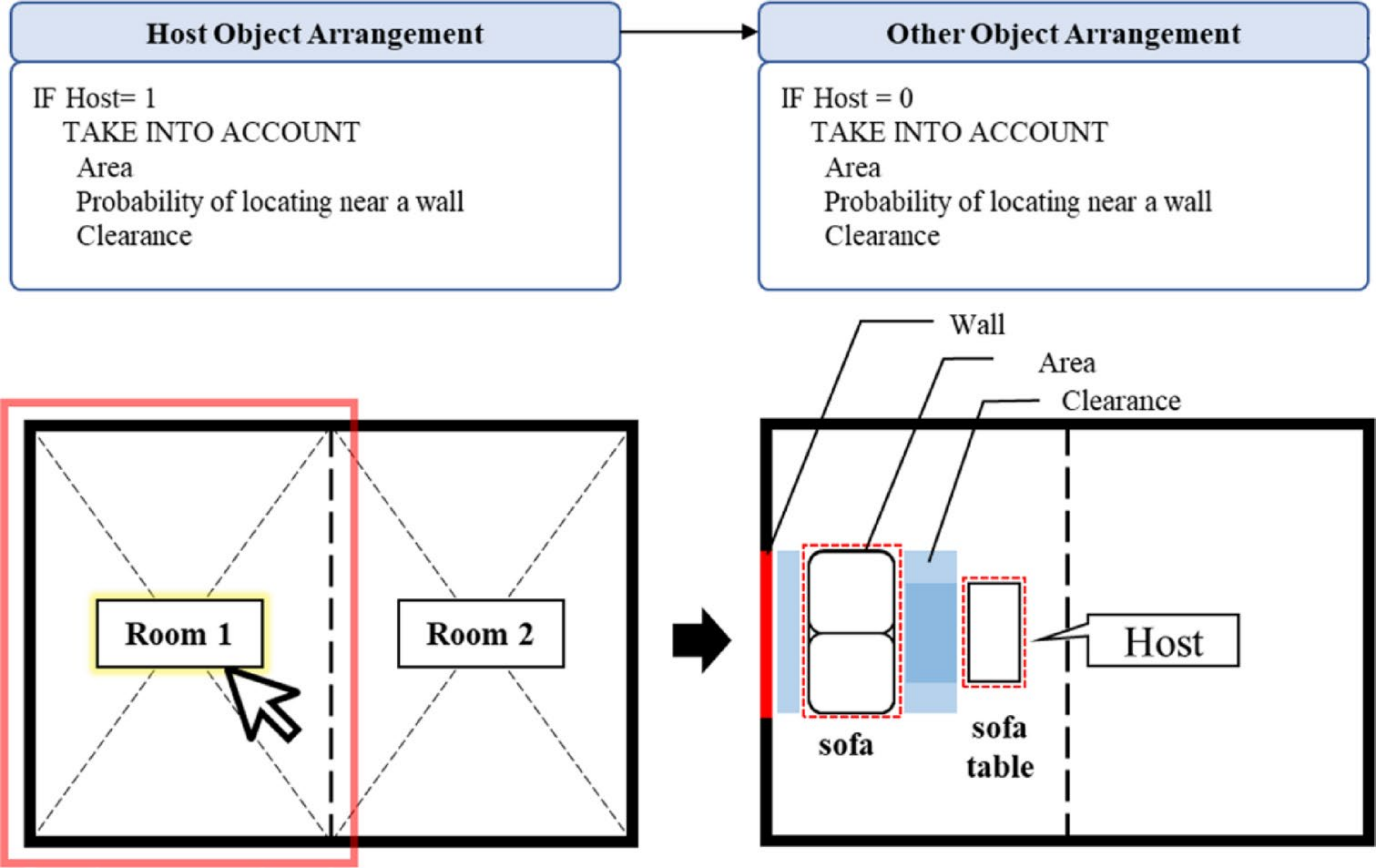
Alignment process considering the host object. A diagram illustrating the two-step placement logic: (1) Host Object Arrangement, placing the primary object (Host = 1) based on its properties (e.g., probability of locating near a wall); (2) Other Object Arrangement, placing child objects (Host = 0) relative to the host, respecting clearance constraints.

‘Arrangement of BIM Composite Object’ phase involves using the attributes for space division, object selection, and object alignment (Fig. 8). The first step is to divide the space into the pathway and actual area to ensure that the furniture does not obstruct the pathway. Next, the user selects the object type, such as a chair, sofa, etc., and the number to be used for BIM composite objects. Finally, in the object alignment step, the selected objects are aligned based on pre-defined rules from design guidelines and object properties. The host object is fixed initially, considering the given attributes, and the other objects are then placed near the host object while taking into account the clearance and object area.

The output phase of the furniture layout results in a preliminary layout, which can be further modified based on external factors such as changes in area or the number of users. Once the changes have been made, the design is finalized, and the furniture layout is generated, including the property window with qualitative information on the BIM composite object.

### Definition of modules

**Fig. 9:**
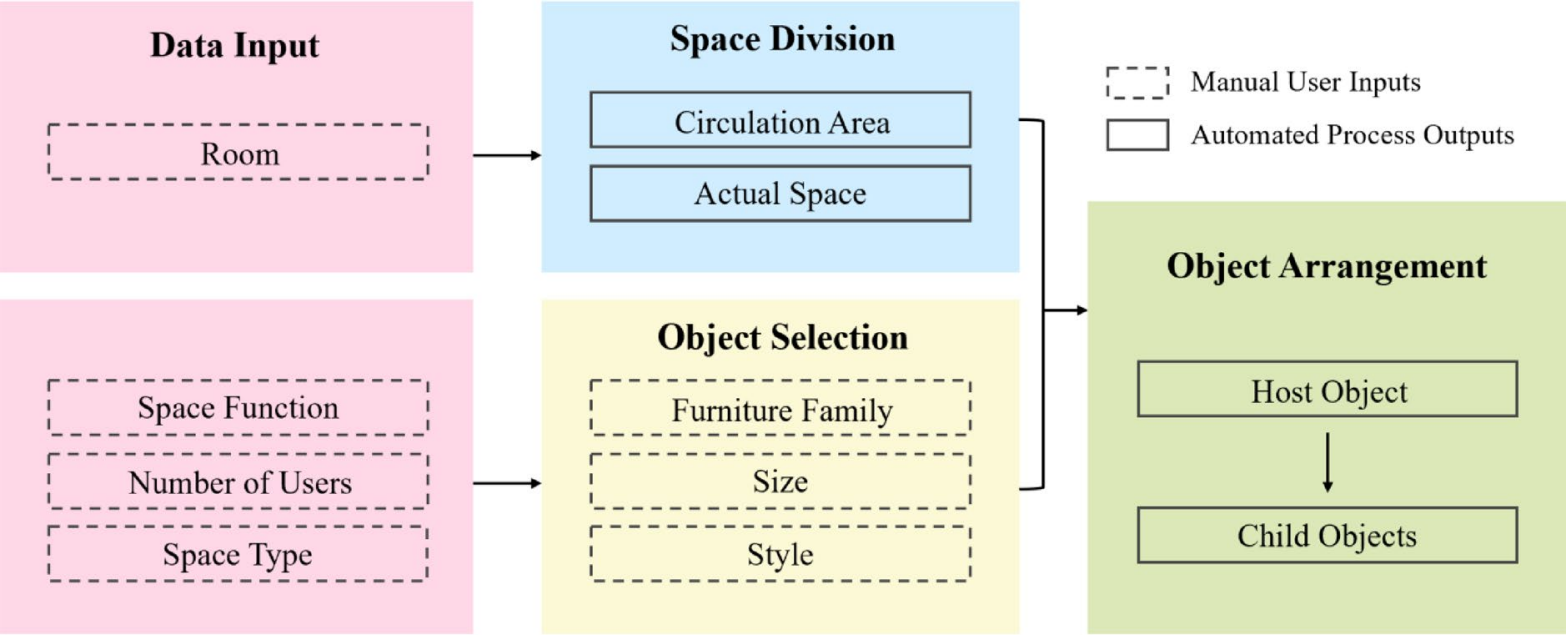
The overall implementation process using Dynamo. A flowchart of the four main modules: (1) Data Input (pink), capturing manual user requirements; (2) Space Division (blue), defining the usable area; (3) Object Selection (yellow), loading the required furniture; (4) Object Arrangement (green), placing objects based on rules. (Dashed-line boxes denote Manual User Inputs; Solid-line boxes denote Automated Process Outputs.).

The process was implemented using Dynamo as shown in Fig. 9. The process comprises four modules: Data Input, Space Division, Object Selection, and Object Arrangement, which are described in detail below.

#### Data input module

**Fig. 10:**
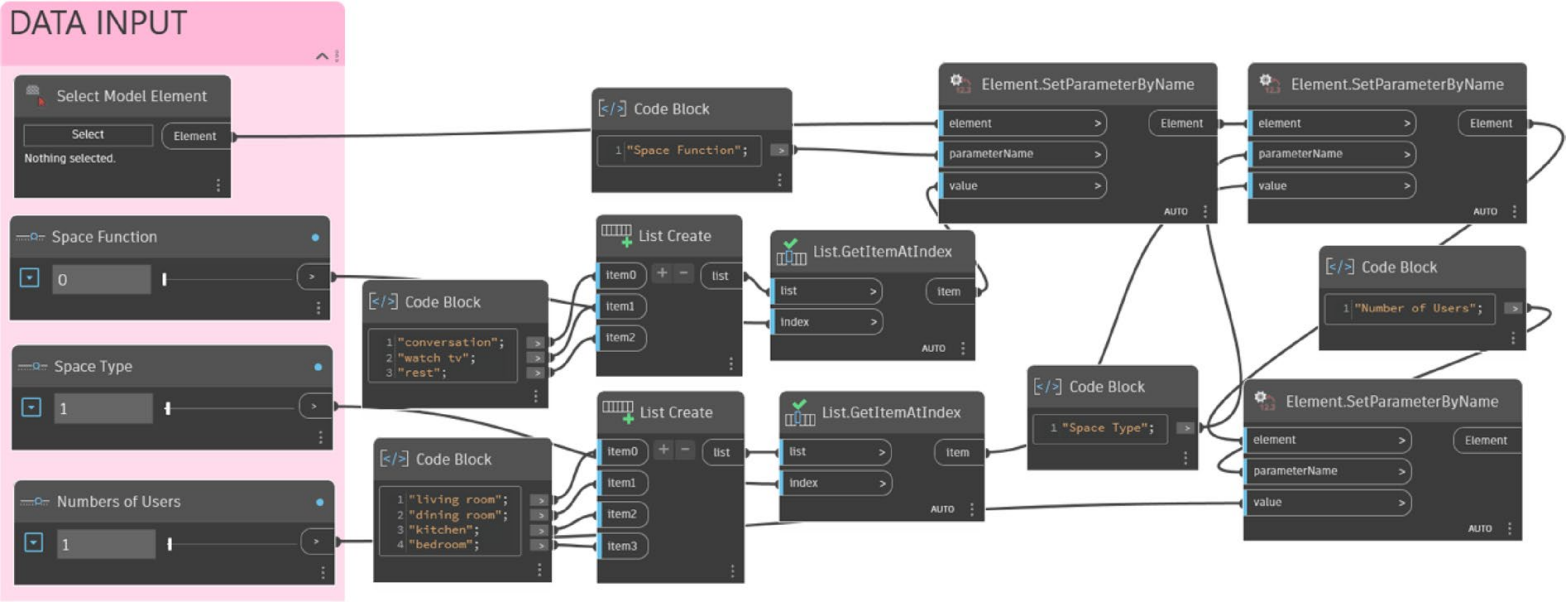
Data input module. The script’s function shown in three parts: (1) Selecting the target ‘Room’ element from the Revit model; (2) Setting key design parameters (e.g., ‘Space Function’) via sliders; (3) Writing these values to the Room object’s properties.

The data input module involves user selections of basic spatial attributes and design requirements (Fig. 10). To obtain the basic spatial attributes, the user selects the target room object in the Revit interface, which provides information such as constraints, dimensions, and identity data. For design requirements, the user selects the space type (e.g., living room, dining room, kitchen), space function (e.g., conversation, watching TV), and the number of users for the target room. The parameters of the target room object are adjusted based on the user inputs and displayed in the property window. The adjusted room information is then exported to the next stage.

#### Space division module

**Fig. 11:**
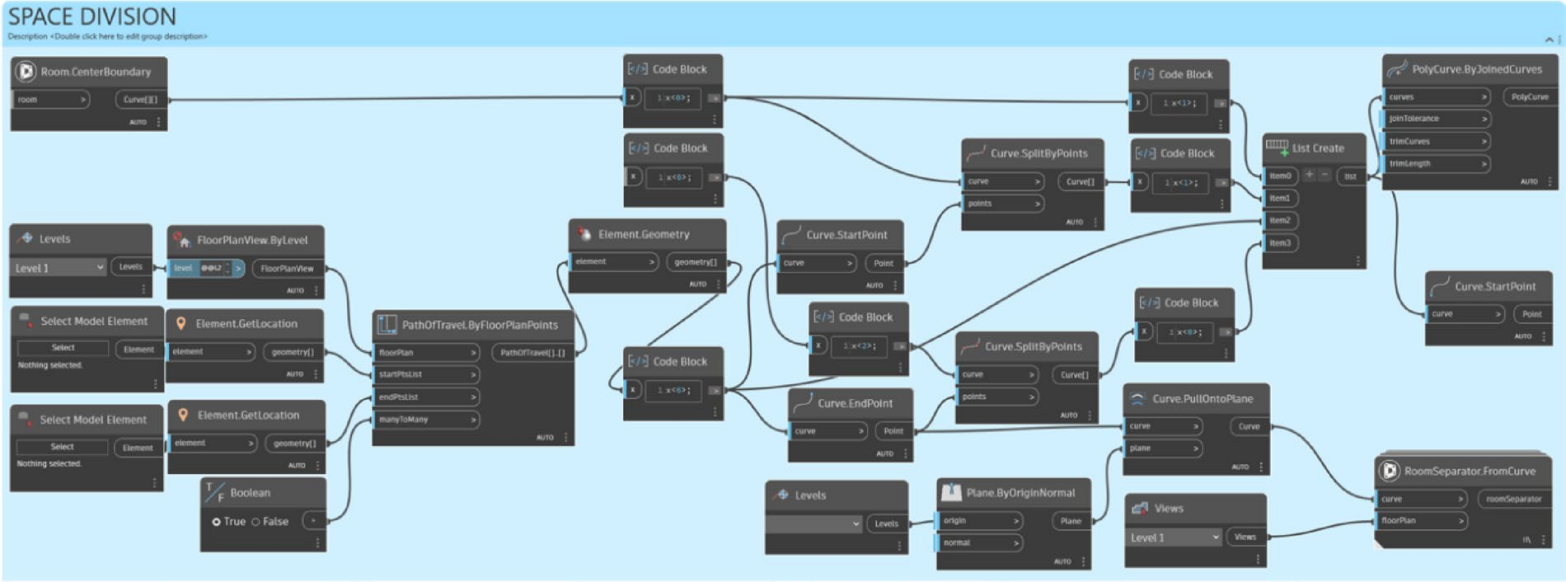
Space division module. A two-step script: (1) Defining circulation paths using Revit’s ‘Path of Travel’ function; (2) Dividing the room’s geometry to create a new ‘Actual Space’ boundary that excludes these pathways.

In this phase, the room object imported from the data input module is utilized (Fig. 11). The room is divided into two parts: the circulation area and the actual space where furniture objects can be placed. The room separation line is created using the *Path of Travel* function in Revit, and the room is cut based on the path to limit it to the actual space. This results in changes to the room object and the adjustment of information such as the room area and boundary line to reflect the actual space that will be used.

#### Object selection module

**Fig. 12:**
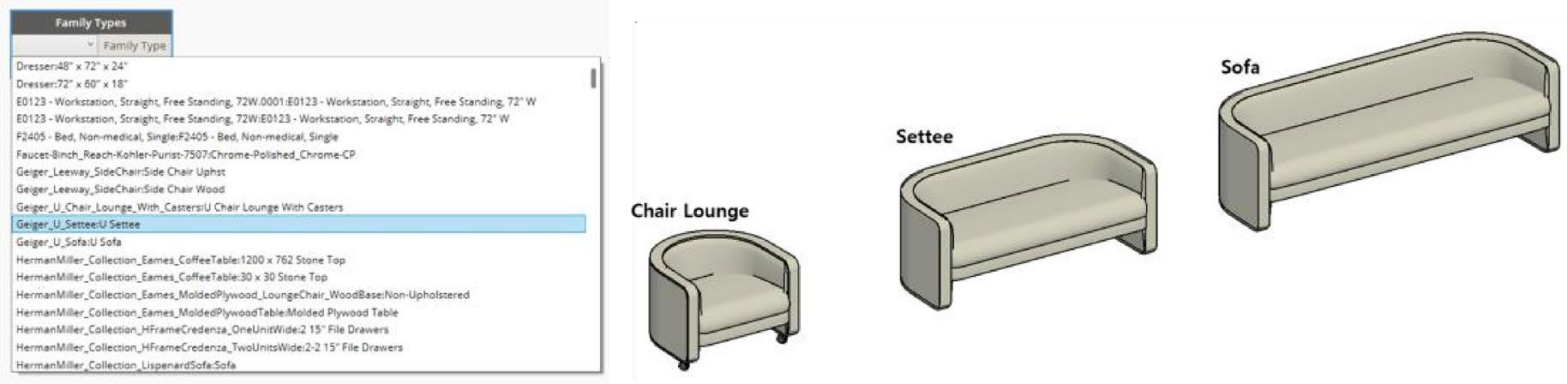
Object selection module. (Left) The user selects a base ‘Family Type’ from the Dynamo interface. (Right) 3D models showing different objects within the same family.

The object selection step allows users to choose their preferred furniture objects in the Dynamo interface. Since the furniture objects have parameters corresponding to the hierarchy, users can select the host object accordingly. The selected object can be used in different sizes or for different numbers of people, depending on the characteristics of the space, such as the area or the number of people. For example, if the *Geiger Lounge chair* is selected, a settee for two or a sofa for three with the same design can be used interchangeably, as shown in Fig. 12.

#### Object arrangement module

**Fig. 13:**
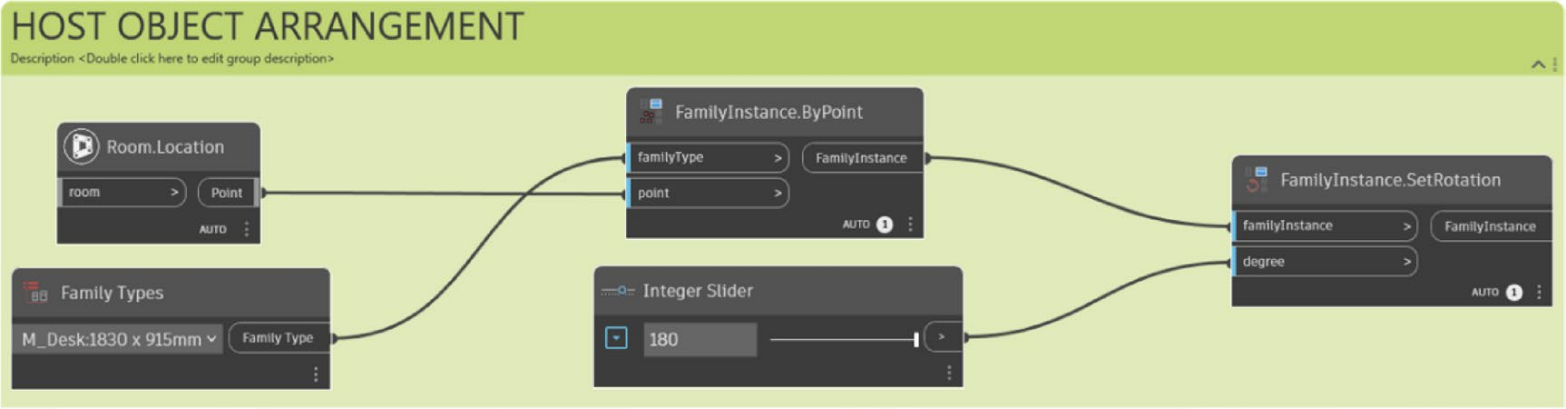
Host object arrangement module. A script for placing the primary ‘Host Object’: (1) Placing the selected furniture family at the room’s location point; (2) Providing user-defined rotation control via an ‘Integer Slider’ to fine-tune the final angle.

**Fig. 14:**
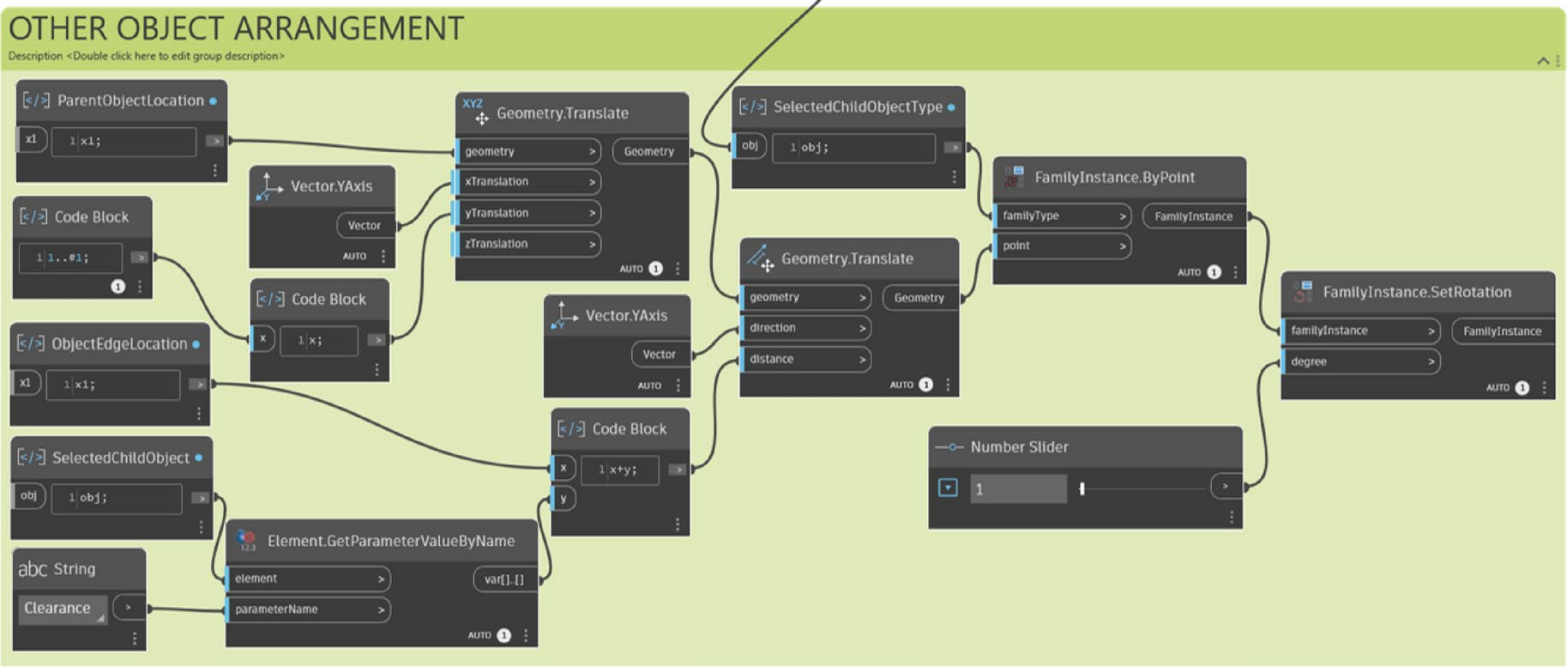
Other object arrangement module. A script for arranging ‘child’ objects relative to the host: (1) Retrieving the host object’s location and geometry; (2) Placing new objects at a specified ‘Clearance’ distance using vector translation.

During the object arrangement stage, the selected host objects are initially positioned in the host object arrangement module (Fig. 13), and then the other objects are arranged relative to the host object in the other object arrangement module (Fig. 14). The placement of the host object is determined based on the space function, either in the center of the room or against the wall. If the host object is placed against the wall, it is positioned with a certain clearance to accommodate the object’s characteristics. The host object’s size or shape may also be adjusted based on the size of the space. Additionally, as seen in Fig. 13, the user can interactively adjust the rotation of the host object using an ‘Integer Slider’ in Dynamo, allowing for fine-tuning of the final layout.

The other object arrangement module places objects based on the location of the host object. It combines the clearance parameter, which is the minimum distance between objects, with the width of the child object, and places the objects in consideration of the distance between them, based on the midpoint of the host object.

## Prototype of residential space by Bim composite objects

### Test scenario setting

**Fig. 15:**
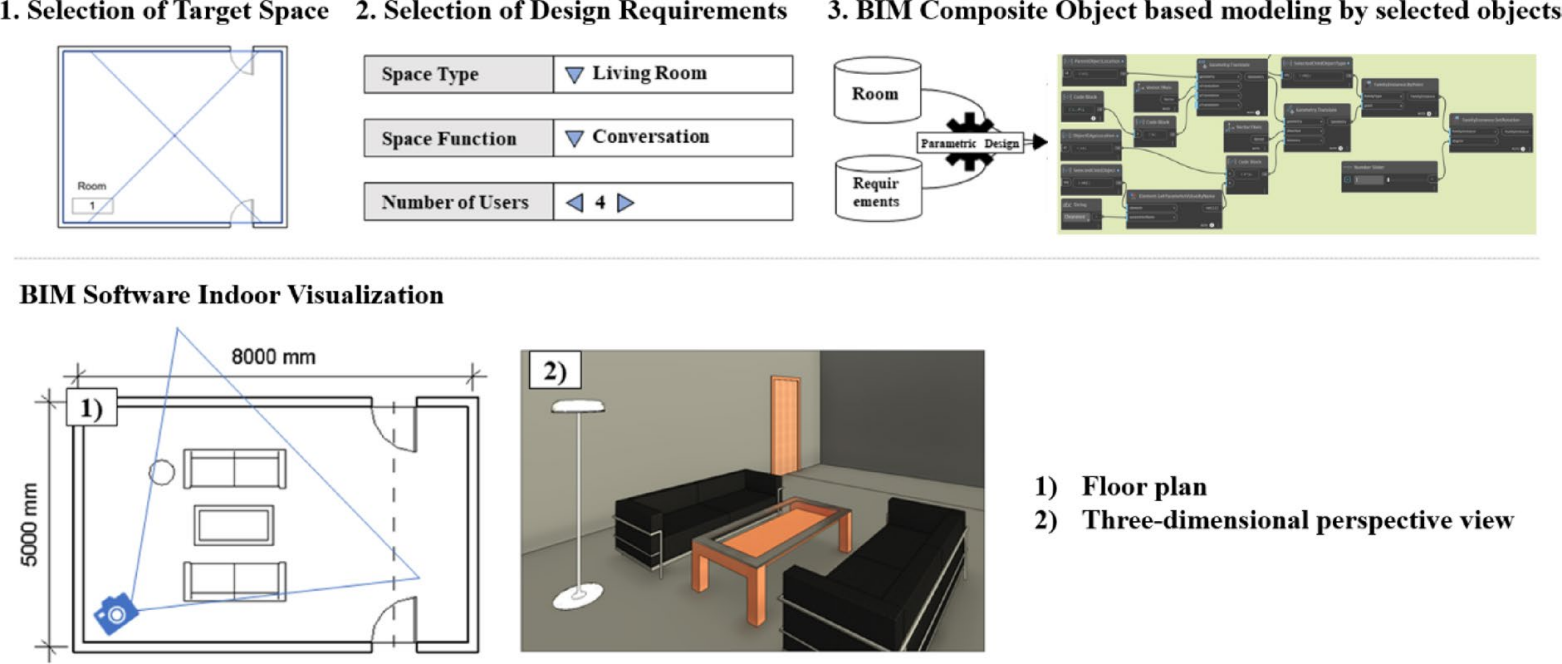
BIM composite object-based space modeling scenario and visualization examples.

**Fig. 16:**
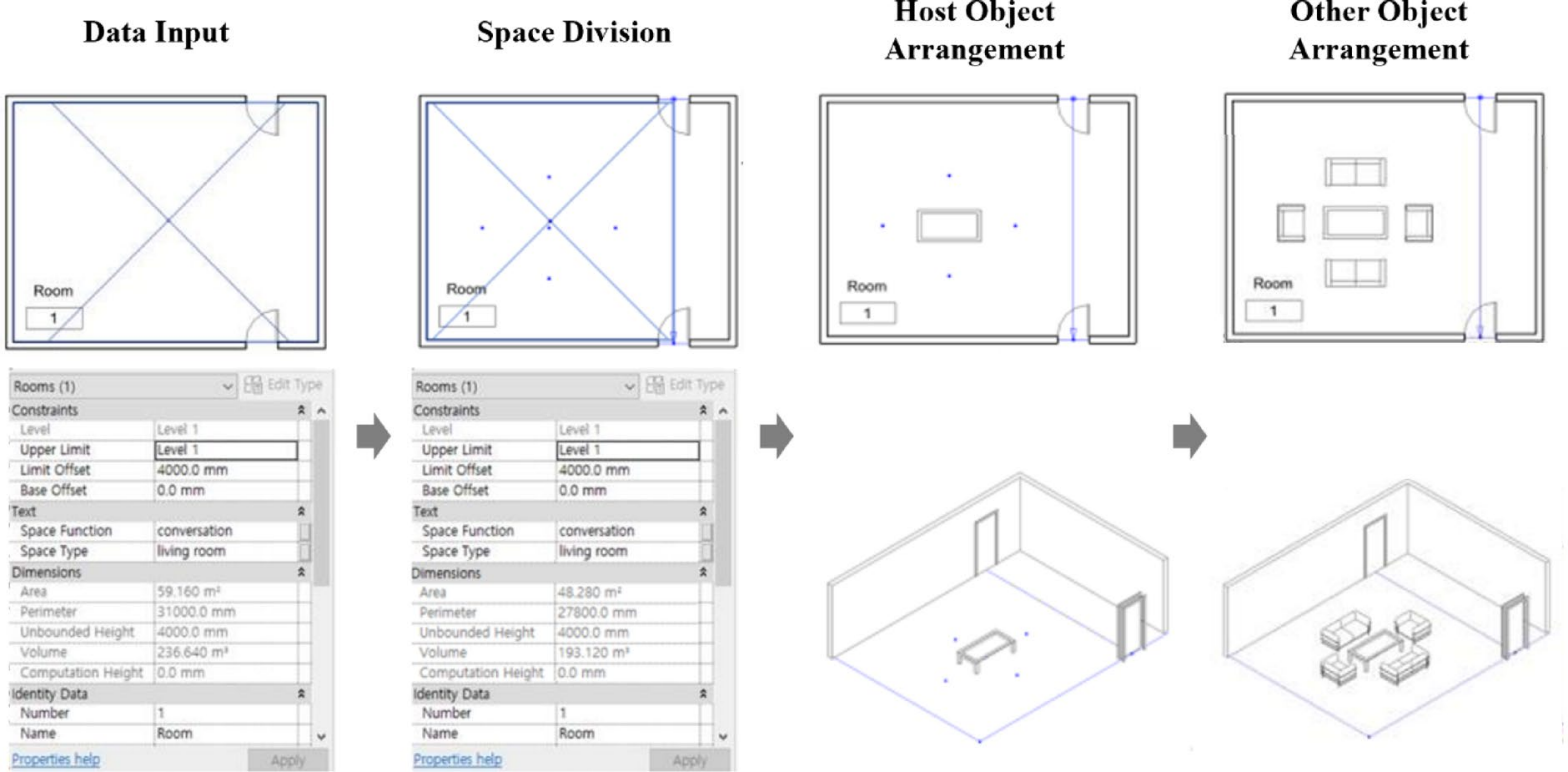
Step-by-step visualization of the automated modeling process. The sequential evolution of a single room layout corresponding to the Dynamo modules: (**a**) Data Input, defining spatial parameters; (**b**) Space Division, calculating the actual usable area; (**c**) Host Object Arrangement, placing the primary anchor furniture; and (**d**) Other Object Arrangement, completing the layout with child objects.

In Seoul, there is an increasing demand for affordable and immediately available urban lifestyle housing for socially vulnerable groups. However, the existing design process is time-consuming, making it difficult to swiftly supply such housing. To address this issue, this study proposes an automated placement method for interior design objects using BIM technology and executes a composite object-based residential space modeling prototype, as shown in Fig. 15, to validate the proposed approach. The study presents various layout options using composite objects based on changes in area, number of users, and space function under the same conditions. Visualization data, such as floor plans and 3D perspectives, are utilized to compare the interior visualizations of these layouts (Fig. 16).

### Results of composite object-based space modeling prototype

The results of the prototype’s four scenarios are presented in Table 4. Scenario A involves a living room for conversation for two people, with a total room area of 27.84 m^2^ and an actual usable area of 20.64 m^2^. The selected objects are a lounge chair, coffee table (serving as the host object), and floor stand. Other objects are arranged based on their relationship with the host object, considering clearance. A minimum gap of 300 mm is required between the lounge chair and coffee table, and the floor stand is placed with a 100 mm gap from surrounding objects.

**Table 4:** Results of the BIM composite object-based space modeling prototype.

**Variables**		**Scenario A**	**Scenario B**	**Scenario C**	**Scenario D**
**Basic Spatial Attribute**	**Name**	Room 1	Room 2	Room 2	Room 2
	**Area**	27.84m^2^	37.44m^2^	37.44m^2^	37.44m^2^
	**Actual area**	20.64m^2^	30.24m^2^	30.24m^2^	30.24m^2^
	**Room shape**				
**Design Requirement**	**Space type**	Living room	Living room	Living room	Living room
	**Space function**	Conversation	Conversation	Watch TV	Conversation and Work
	**Number of users**	2	4	4	4
**Object Selection**	**Selected object**				
	**Host object**	Coffee table	Coffee table	TV	Desk
**Object Arrangement**	**Clearance between objects**	- Chair lounge/Sofa-Coffee table: More than 300mm - Floor stand: More than 100mm	- Chair lounge/Sofa-Coffee table: More than 300mm - Floor stand: More than 100mm	- TV-Sofa: More than 2440mm - Chair Lounge-Sofa (90**°** arrangement): More than 100mm - Chair lounge/Sofa-Coffee table: More than 300mm	- Desk-Coffee table: More than 1200mm - Chair lounge/Sofa-Coffee table: More than 300mm
	**Floorplan**				
	**3D Perspective View**				

In Scenario B, the total area is 37.44 m^2^, 2 m wider than in Scenario A, with 30.24 m^2^ available for object placement. Designed for conversation, the selected objects are a sofa, coffee table (the host object), and floor stand, with other objects placed based on the coffee table’s location. Clearance between objects is considered during placement.

Scenario C shares the same total area as Scenario B (37.44 m^2^ with 30.24 m^2^ for object placement) but is intended for television viewing. The selected objects are a lounge chair, sofa, coffee table, and television—the host object—arranged for optimal viewing. A distance of 2440 mm is needed between the television and sofa for a 60-inch screen. The lounge chair, sofa, and coffee table are placed with minimum gaps of 300 mm, and when arranged at a right angle, a minimum gap of 100 mm is necessary. These distances ensure comfort, functionality, and safety for users.

Scenario D addresses the growing trend of home offices post-COVID-19, integrating workspaces within residential areas. A living room with the same spatial attributes as Scenario B is designed to accommodate both conversation and work. The selected objects are a sofa, coffee table, desk (the host object), and chair. The desk is strategically positioned facing the wall to enhance focus, with a minimum distance of 1200 mm between the desk and coffee table to allow comfortable passage. The chair is placed close to the desk within this distance, and the sofa is at least 300 mm from the coffee table, as in Scenario B.

Scenarios A and B differ in the size and number of objects due to differences in area and occupancy, but object types remain constant because of the same space type and function. To maintain a consistent ratio between household and space area, the size of the sofa or table can be modified without adding more objects. In Scenario A, the furniture area accounts for 10.2% of the space area; in Scenario B, it accounts for approximately 11.2%, indicating a similar ratio despite changes in occupancy.

In contrast, Scenarios B, C, and D have the same space area and number of users but differ in function, resulting in changes in available objects. In Scenario B, the table is the host object due to having the most surrounding objects. In Scenario C, the addition of a fixed television with a low movability parameter changes the host object. Scenario D enhances Scenario B by adding a desk to accommodate work, shifting the host object to the desk, similar to the television in Scenario C. This addition changes the space’s primary focus from social interaction to include work activities.

However, it is important to note the limitations of the current algorithm. The successful scenarios were conducted in simple, rectangular rooms. To test the system’s robustness, an exploratory test was performed in a non-rectangular, L-shaped space. In this case, the algorithm, which assumes a convex spatial boundary, failed to correctly place objects, resulting in furniture intersecting with a wall. This failure case indicates that the current space division logic needs further development to handle more complex, non-convex geometries.

### Quantitative evaluation: focus group interview (FGI)

To validate the proposed method’s efficiency and practicality, we conducted a Focus Group Interview (FGI) with five distinct experts in the field of BIM and architectural design. To ensure the reliability of the evaluation, the participants were selected based on their professional expertise and practical experience. The panel consisted of two professors from accredited architectural programs, a CEO and a Senior Software Developer (with 15 years of experience) from a specialized BIM software development firm, and a Ph.D. candidate with 5 years of professional industry experience.

The evaluation consisted of two sessions: (1) a comparative modeling efficiency test and (2) a qualitative survey on design satisfaction and usability.

For modeling efficiency test, participants were tasked with modeling the four scenarios (A, B, C, D) defined in Sect. 5.1 using both the traditional manual method and the proposed automated tool. The Manual method involved selecting families, placing them, and adjusting their rotation and clearance individually to meet the design rules. The Automated method involved inputting parameters into the Dynamo interface and generating the layout.

As shown in Table 5, the results indicate a dramatic reduction in modeling time. The average total time to complete all four scenarios manually was 47.2 min, whereas the automated approach took only 10.4 min. This corresponds to an average time saving of 78.0%.

**Table 5:** Comparison of modeling times between manual and automated approaches (*n* = 5).

Participant	Position	Total Manual Time (min)	Total Automated Time (min)	Time Savings (%)
Expert 1	Professor (Interior Arch.)	48.5	11.0	77.3
Expert 2	Professor (Architecture)	50.0	12.5	75.0
Expert 3	CEO (BIM Firm)	42.5	8.0	81.2
Expert 4	Senior Software Developer	45.0	9.5	78.9
Expert 5	Ph.D. Candidate (5y exp)	50.0	11.0	78.0
Average	-	47.2	10.4	78.0

Following the practical test, a survey was conducted to assess the quality of the generated layouts and the usability of the tool. The survey utilized a 5-point Likert scale (1 = Strongly Disagree, 5 = Strongly Agree). The protocol and summarized results are presented in Table 6.

The average score for ‘Design Satisfaction’ was 4.2, indicating that the experts found the automated layouts to be functionally appropriate and compliant with design rules. The ‘Tool Ease of Use’ score was 4.6, suggesting that the Dynamo interface was intuitive even for those not specialized in coding. Moreover, the design rule compliance rate was rated a perfect 5.0 (100%) across all scenarios, proving that the system operates accurately according to the constraints defined in Table 3.

**Table 6:** Survey protocol and expert evaluation results (*n* = 5).

Category	Question Item	Scale	Average Score
Design Satisfaction	Q1. How satisfied are you with the quality and functional appropriateness of the automatically generated layouts?	a 5-point Likert scale (1 = Strongly Disagree, 5 = Strongly Agree	4.2
Tool Ease of Use	Q2. How would you rate the ease of use of the automated tool compared to the manual modeling process?		4.6
Rule Compliance	Q3. Did the automated results strictly follow the given design rules (e.g., Clearance ≥ 300 mm)?		5.0

## Conclusion

### Summary and contributions

This study presents a methodology utilizing BIM composite objects for efficient indoor space modeling. By adopting a space-centered perspective instead of the traditional object-oriented approach, the methodology aims to improve modeling efficiency. The term “BIM composite object” is defined along with its components and functionality. To achieve this, space variables and object properties are pre-established using design guidelines from interior architecture and prior research on automated furniture layout generation.

The main contributions are: Firstly, the study proposes a space-centered approach that decomposes large spaces into smaller, manageable units using BIM composite objects. This method contrasts with the traditional object-oriented approach and offers a more efficient modeling process. Secondly, the introduction of BIM composite objects as a novel concept with clearly defined composition and functionality enables more effective modeling by providing predefined space variables and object properties. Lastly, the study presents a three-step methodology for residential space modeling using BIM composite objects: data input, composite object arrangement, and furniture layout output, resulting in a more systematic and streamlined modeling process.

By introducing BIM composite objects and automating the furniture layout process, the study aims to increase efficiency for designers. With the proposed method, designers input basic spatial and user attributes, and the software automatically generates furniture layouts based on predefined design guidelines. This approach reduces redundancy, enabling designers to focus on more complex and creative tasks, which ultimately leads to higher productivity and improved design outcomes^58–61^.

### Limitations and future works

Despite its contributions, this study has several limitations that open avenues for future research. First, the prototype is limited to living rooms and has not been validated for other space types with stricter ergonomic constraints, such as kitchens (e.g., the work triangle) or offices. Second, the current system operates in simplified rectangular rooms and does not consider critical architectural constraints such as the location of windows, heating elements, or electrical outlets. Third, the rule set does not yet incorporate accessibility guidelines, such as ensuring sufficient turning radii for wheelchairs. Fourth, the sets of attributes and rules presented (e.g., in Fig. 6; Table 3) are foundational rather than exhaustive and can be expanded in future research to include a wider range of design constraints. Currently, the rule set focuses on quantifiable metrics (e.g., distance, angle) and does not yet fully capture subjective design principles such as aesthetic harmony or style coherence. Finally, the furniture objects are selected manually by the user; a function to automatically recommend furniture that matches the space’s style has not been implemented.

Future work will focus on overcoming these limitations by developing rule sets applicable to diverse space types, integrating architectural and accessibility constraints, and enhancing the system to support non-rectangular spaces. Furthermore, we plan to incorporate machine learning to recommend furniture based on user style preferences, evolving the system into a more intelligent design support tool.

## Data Availability

The datasets used and/or analysed during the current study are available from the corresponding author on reasonable request.

## References

[1] Park, S. & Lee, J. K. A study on the technological connections between BIM (building information modeling) and interior architecture design – focusing on the applications of Spatial object and its properties. *J. Korean Des. Knowl.* **34**, 35–44. 10.17246/jkdk.2015.34.004 (2015).

[2] Eastman, C. Automated assessment of early concept designs. *Architectural Des.* **79** (2), 52–57. 10.1002/ad.851 (2009).

[3] Lee, J. K. et al. Development of space database for automated Building design review systems. *Autom. Constr.* **24**, 203–212. 10.1016/j.autcon.2012.03.002 (2012).

[4] Alawadhi, M. & Yan, W. Deep learning from parametrically generated virtual buildings for real-world object recognition. ArXiv Preprint. arXiv:2302.05283. (2023). 10.48550/arXiv.2302.05283

[5] Fisher, M., Ritchie, D., Savva, M., Funkhouser, T. & Hanrahan, P. Example-based synthesis of 3D object arrangements. *ACM Trans. Graphics*. **31.6**, 1–11. 10.1145/2366145.2366154 (2012).

[6] Ustyugov, A. How will artificial intelligence change our living space? Forbes. (2021). https://www.forbes.com/sites/forbestechcouncil/2021/09/30/how-will-artificial-intelligence-change-our-living-spaces/?sh=753fa54e20b6

[7] Kim, J. & Lee, J. K. Stochastic detection of interior design styles using a deep-learning model for reference images. *Appl. Sci.* **10**, 20, 7299. 10.3390/app10207299 (2020).

[8] Baduge, S. et al. Artificial intelligence and smart vision for Building and construction 4.0: machine and deep learning methods and applications. *Autom. Constr.* **141**, 104440. 10.1016/j.autcon.2022.104440 (2022).

[9] Onur, N. A review of the use of examples for automating architectural design tasks. *Comput. Aided Des.* **96**, 13–30. 10.1016/j.cad.2017.10.005 (2018).

[10] Pan, Y., Zhang, L. & Integrating BIM and AI for smart construction management: current status and future directions. *Arch. Comput. Methods Eng.* **30**, 1081–1110. 10.1007/s11831-022-09830-8 (2023).

[11] Pizarro, P. N., Hitschfeld, N., Sipiran, I. & Saavedra, J. M. Automatic floor plan analysis and recognition. *Autom. Constr.* **140**, 104348. 10.1016/j.autcon.2022.104348 (2022).

[12] Regona, M. et al. Opportunities and adoption challenges of AI in the construction industry: A PRISMA review. *J. Open. Innovation: Technol. Market Complex.* **8** (1), 45. 10.3390/joitmc8010045 (2022).

[13] Samuel, A., Mahanta, N. R. & Technologies, N. Casel Vitug, A. Computational technology and artificial intelligence (AI) revolutionizing interior design graphics and modelling. 13th International Conference on Computing Communication and (ICCCNT), 1–6. (2022). 10.1109/ICCCNT54827.2022.9984232

[14] Waayenberg, E. The future of design: Technology, automation, and how we should respond. Interior Design. (2021). https://interiordesign.net/designwire/the-future-of-design-technology-automation-and-how-we-should-respond/

[15] Wang, H. et al. BIM-based automated design for HVAC system of office buildings—An experimental study. *Build. Simul.* **15**, 1177–1192. 10.1007/s12273-021-0883-7 (2022).

[16] Zhang, F., Chan, A. P., Darko, A., Chen, Z. & Li, D. Integrated applications of Building information modeling and artificial intelligence techniques in the AEC/FM industry. *Autom. Constr.* **139**, 104289. 10.1016/j.autcon.2022.104289 (2022).

[17] Hong, S. et al. Evaluation of practical requirements for automated detailed design module of interior finishes in architectural Building information model. *Korean J. Constr. Eng. Manage.* **23** (5), 87–97. 10.6106/KJCEM.2022.23.5.087 (2022).

[18] Wang, K. et al. Planning and instantiating indoor scenes with relation graph and Spatial prior networks. *ACM Trans. Graphics*. **38** (4), 1–15. 10.1145/3306346.3322941 (2019).

[19] Lee, J. K. & Kim, M. BIM-enabled conceptual modelling and representation of Building circulation. *Int. J. Adv. Rob. Syst.*10.5772/58440 (2014). 11.127.

[20] Lee, Y., Eastman, C. & Lee, J. K. Validations for ensuring the interoperability of data exchange of a building information model. *Autom. Constr.* **58**, 176–195. 10.1016/j.autcon.2015.07.010 (2015).

[21] Uhm, M. et al. Requirements for computational rule checking of requests for proposals (RFPs) for Building designs in South Korea. *Adv. Eng. Inform.* **29.3**, 602–615. 10.1016/j.aei.2015.05.006 (2015).

[22] Park, D. & Cha, H. A developing a machine leaning-based defect data management system for multi-family housing unit. *Korean J. Constr. Eng. Manage.* **24** (5), 35–43. 10.6106/KJCEM.2023.24.5.035 (2023).

[23] Seoul Housing Portal. Housing policy for urban living housing. (2023). https://housing.seoul.go.kr/site/main/content/sh01_030800

[24] Choi, S., Kim, Y., Nam, T., Hong, S. W. & Lee, J. K. Generative architectural plan drawings for early design decisions: Data grounding and additional training for specific use cases. *Architectural Eng. Des. Manage.*, 1–21. 10.1080/17452007.2024.2445033 (2024).

[25] Park, Y. & Oh, H. Interior design: Theory and for housing plan and realityDaseossure,. (1993).

[26] Baek, H. S., Choi, S. H., Choi, D. S. & Joo, J. Y. Planning design guidelines for LH unit plan. *Land. Hous. Inst.* **16** (12), 71–82 (2012).

[27] Autodesk. Revit for architecture & building design. https://www.autodesk.com/products/revit/overview

[28] Autodesk. Dynamo for Revit. https://www.dynamobim.org/dynamo-for-revit/

[29] Merrell, P., Schkufza, E., Li, Z., Agrawala, M. & Koltun, V. Interactive furniture layout using interior design guidelines. *ACM Trans. Graphics*. **30** (4), 1–10. 10.1145/1964921.1964982 (2011).

[30] Jiang, S., Wang, M. & Ma, L. Gaps and requirements for applying automatic architectural design to Building renovation. *Autom. Constr.* **147**, 104742. 10.1016/j.autcon.2023.104742 (2023).

[31] Sydora, C. & Stroulia, E. Rule-based compliance checking and generative design for Building interiors using BIM. *Autom. Constr.* **120**, 103368. 10.1016/j.autcon.2020.103368 (2020).

[32] Yu, L. et al. Make it home: automatic optimization of furniture arrangement. *ACM Trans. Graphics*. **30** (4), 1–12. 10.1145/2010324.1964981 (2011).

[33] Kán, P. & Kaufmann, H. Automated interior design using a genetic algorithm. 23rd ACM Symposium on Virtual Reality Software and Technology 25, 1–10. (2017). 10.1145/3139131.3139135

[34] Lee, J. K., Eastman, C. M., Lee, J., Kannala, M. & Jeong, Y. S. Computing walking distances within buildings using the universal circulation network. *Environ. Plan.* **37** (4), 628–645. 10.1068/b35124 (2010).

[35] Zhang, S. H., Zhang, S. K., Xie, W. Y., Luo, C. Y. & Fu, H. B. Fast 3D indoor scene synthesis with discrete and exact layout pattern extraction. ArXiv Preprint. arXiv:2002.00328. (2020). 10.48550/arXiv.2002.00328

[36] Zhang, Z. et al. Deep generative modeling for scene synthesis via hybrid representations. *ACM Trans. Graphics*. **39** (2), 1–21. 10.1145/3381866 (2020).

[37] Lee, K. J. & Kim, S. G. *Housing Planning Theory* (Bosungak, 2001).

[38] Kim, H. D. *Housing Plan Design* (Kimoondang, 1999).

[39] Kang, B. S. et al. *History of Korean Apartment Housing Planning* (Sejinsa, 1999).

[40] Pile, J. F. *Interior design* (Harry N. Abrams, 1988).

[41] Panero, J. & Zelnik, M. *Human dimension & interior space: a source book of design reference standards* (Whitney Library of Design, 1979).

[42] Tilley, A. R. & Henry Dreyfuss Associates. &. The measure of man and woman: human factors in design, revised edition. (John Wiley & Sons, (2001).

[43] Neufert, E. & Neufert, P. *Architects’ data* (Wiley-Blackwell, 2012).

[44] Peterson, C. *Home office solutions: how to set up an efficient workspace anywhere in your house* (Fox Chapel Publishing, 2020).

[45] Stability, A. I. Stable Diffusion. (2022). https://stability.ai/blog/stable-diffusion-public-release

[46] Finch Finch 3D. (2020). https://finch3d.com/.

[47] Autodesk Revit Generative Design. (2020). https://www.autodesk.com/solutions/generative-design/architecture-engineering-construction

[48] Testfit TestFit. (2017). https://testfit.io/.

[49] Autodesk. Spacemaker, A. I. (2018). https://www.autodesk.com/products/spacemaker/overview

[50] Jo, H., Lee, J. K., Lee, Y. C. & Choo, S. Generative artificial intelligence and Building design: early photorealistic render visualization of façades using local identity-trained models. *J. Comput. Des. Eng.* **11** (2), 85–105. 10.1093/jcde/qwae017 (2024).

[51] Robert, McNeel & Associates Rhinoceros. https://www.rhino3d.com/

[52] Robert, McNeel & Associates Grasshopper. https://www.grasshopper3d.com/

[53] buildingSMART & International ISO 16739-1:2018 Industry foundation classes (IFC) for data sharing in the construction and facility management industries — Part 1: Data schema. (2018). https://technical.buildingsmart.org/standards/ifc/

[54] Kim, K. & Yu, J. Integrated information management for composite object properties in BIM. *Korea Inst. Constr. Eng. Manage.* **16** (2), 97–105. 10.6106/KJCEM.2015.16.2.097 (2015).

[55] Ma, R. et al. Language-driven synthesis of 3D scenes from scene databases. *ACM Trans. Graphics*. **37** (6), 1–16. 10.1145/3272127.3275035 (2018).

[56] Zhang, S., Teizer, J., Lee, J. K., Eastman, C. M. & Venugopal, M. Building information modeling (BIM) and safety: automatic safety checking of construction models and schedules. *Autom. Constr.* **29**, 183–195. 10.1016/j.autcon.2012.05.006 (2013).

[57] Zhao, Q., Zhou, L., Lv, G. & Computers A 3D modeling method for buildings based on LiDAR point cloud and DLG. *Environ. Urban Syst.* **102**, 101974. 10.1016/j.compenvurbsys.2023.101974 (2023).

[58] Karan, E. & Asadi, S. Intelligent designer: A computational approach to automating design of windows in buildings. *Autom. Constr.* **102**, 160–169. 10.1016/j.autcon.2019.02.019 (2019).

[59] Song, J. & Lee, J. K. An approach to implementing automated modeling of interior design object using Spatial information training model - focused on an implementation example of automated layout system for ceiling light object. *J. Korean Inst. Interior Des.* **29** (2), 12–20. 10.14774/JKIID.2020.29.2.012 (2020).

[60] Sanguinetti, P. et al. General system architecture for BIM: an integrated approach for design and analysis. *Adv. Eng. Inform.* **26.2**, 317–333. 10.1016/j.aei.2011.12.001 (2012).

[61] Zhao, P., Liao, W., Xue, H. & Lu, X. Intelligent design method for beam and slab of shear wall structure based on deep learning. *J. Building Eng.* **57**, 104838. 10.1016/j.jobe.2022.104838 (2022).

